# P-1731. Strengthening Fungal Infection Diagnosis And Treatment: An In-Depth Analysis Of Capabilities In Honduras

**DOI:** 10.1093/ofid/ofaf695.1902

**Published:** 2026-01-11

**Authors:** Jon Salmanton-Garcia, Diana Varela, Gustavo Fontecha, karla torres, Oliver A Cornely, bryan ortiz

**Affiliations:** University Hospital Cologne, Cologne, Germany, Cologne, Nordrhein-Westfalen, Germany; 2. Servicio de Infectología, Servicio de Atención Integral de Pacientes con VIH, Hospital Escuela, Tegucigalpa, Honduras, tegucigalpa, Francisco Morazan, Honduras; 1. Instituto de Investigaciones en Microbiología, Facultad de Ciencias, Universidad Nacional Autónoma de Honduras, Tegucigalpa, Honduras, tegucigalpa, Francisco Morazan, Honduras; 4. Agrupación de Microbiólogos Propietarios de Laboratorios Privados de Honduras (AMIPROLABPH), Tegucigalpa, Honduras, tegucigalpa, Francisco Morazan, Honduras; University of Cologne, Faculty of Medicine and University Hospital Cologne, Cologne, Nordrhein-Westfalen, Germany; 1. Instituto de Investigaciones en Microbiología, Facultad de Ciencias, Universidad Nacional Autónoma de Honduras, Tegucigalpa, Honduras, tegucigalpa, Francisco Morazan, Honduras

## Abstract

**Background:**

Invasive fungal infections (IFI) pose a major public health challenge in low- and middle-income countries (LMICs) due to limited diagnostic and treatment resources, resulting in high morbidity and mortality. Despite their global burden, IFIs remain under-recognized and under-diagnosed in LMICs. This study assesses Honduras' capacity to diagnose and treat IFIs amid its distinct healthcare challenges.

**Methods:**

From March to December 2023, a comprehensive survey was conducted across multiple healthcare centers in Honduras. The survey, reviewed for content and clarity by local medical institutions, targeted medical microbiologists and clinicians to assess various aspects of fungal disease diagnosis and treatment. Data collected included the availability and use of diagnostic tools and antifungal therapies, identifying gaps and limitations in current practices.

**Results:**

The survey revealed that *Candida* spp. (97.4%) and *Aspergillus* spp. (35.9%) were the most concerning pathogens. Although microscopy and culture methods were available in most institutions, their application in suspected IFI cases was inconsistent, and antifungal susceptibility testing was rarely performed. Advanced diagnostic techniques, such as antigen detection, were available in only a few institutions, while antibody detection and PCR testing were entirely absent. All hospitals had access to at least one triazole antifungal, typically fluconazole, but there was a notable scarcity of more potent antifungals, including amphotericin B formulations and echinocandins. The limited use of available diagnostic tools and the restricted availability of essential antifungals were identified as major barriers to effective IFI management.

**Conclusion:**

This study reveals major gaps in Honduras' capacity to diagnose and treat IFIs, including limited use of basic diagnostics, lack of advanced testing, and scarce access to antifungal medications. These deficiencies highlight the urgent need for capacity-building, infrastructure upgrades, and policy reforms. The findings can guide targeted interventions and resource allocation to improve IFI outcomes in Honduras and similar LMICs.

**Disclosures:**

Jon Salmanton-Garcia, MSc, MPH, PhD, menarini, gilead, astrazeneca, pfizer: Honoraria Oliver A. Cornely, Prof. Dr., Al-Jazeera Pharmaceuticals/Hikma: Honoraria|Basilea: Advisor/Consultant|Cidara: Advisor/Consultant|Cidara: Board Member|Cidara: Grant/Research Support|Elion: Advisor/Consultant|F2G: Grant/Research Support|Gilead: Advisor/Consultant|Gilead: Grant/Research Support|Gilead: Honoraria|GlaxoSmithKline: Advisor/Consultant|GlaxoSmithKline: Honoraria|Grupo Biotoscana/United Medical/Knight: Honoraria|Melinta: Advisor/Consultant|Melinta: Board Member|MSD: Honoraria|Mundipharma: Advisor/Consultant|Mundipharma: Grant/Research Support|Mundipharma: Honoraria|Pfizer: Advisor/Consultant|Pfizer: Grant/Research Support|Pfizer: Honoraria|Pulmocide: Board Member|Scynexis: Advisor/Consultant|Scynexis: Grant/Research Support|Shionogi: Advisor/Consultant|Shionogi: Honoraria

